# Sulfated Polysaccharide from *Caulerpa racemosa* Attenuates the Obesity-Induced Cardiometabolic Syndrome via Regulating the PRMT1-DDAH-ADMA with mTOR-SIRT1-AMPK Pathways and Gut Microbiota Modulation

**DOI:** 10.3390/antiox12081555

**Published:** 2023-08-03

**Authors:** Nelly Mayulu, William Ben Gunawan, Moon Nyeo Park, Sanghyun Chung, Jin Young Suh, Hangyul Song, Rio Jati Kusuma, Nurpudji Astuti Taslim, Rudy Kurniawan, Felicia Kartawidjajaputra, Fahrul Nurkolis, Bonglee Kim

**Affiliations:** 1 Department of Nutrition, Faculty of Health Science, Muhammadiyah Manado University, Manado 95249, Indonesia; nmayulu@unsrat.ac.id; 2 Alumnus of Nutrition Science, Faculty of Medicine, Diponegoro University, Semarang 50275, Indonesia; 3 Department of Pathology, College of Korean Medicine, Kyung Hee University, Hoegidong Dongdaemun-gu, Seoul 02447, Republic of Korea; 4 Korean Medicine-Based Drug Repositioning Cancer Research Center, College of Korean Medicine, Kyung Hee University, Seoul 02447, Republic of Korea; 5 Kyung Hee Myungbo Clinic of Korean Medicine, Hwaseong-si 18466, Gyeonggi-do, Republic of Korea; 6 Seoul Forest Korean Medicine Clinic, Ttukseomro 312, Seongdonggu, Seoul 04773, Republic of Korea; 7 Nneul 365 Korean Medical Clinic, 3F, 8-13, Haneulbyeolbit-Ro 65 Beongil, Jung-gu, Incheon 22397, Gyeonggi-do, Republic of Korea; 8 Department of Nutrition and Health, Faculty of Medicine, Public Health, and Nursing, Universitas Gadjah Mada, Yogyakarta 55223, Indonesia; 9 Center of Herbal Medicine, Faculty of Medicine, Public Health, and Nursing, Universitas Gadjah Mada, Yogyakarta 55223, Indonesia; 10 Division of Clinical Nutrition, Department of Nutrition, Faculty of Medicine, Hasanuddin University, Makassar 90245, Indonesia; pudji_taslim@yahoo.com; 11 Alumnus of Internal Medicine, Faculty of Medicine, University of Indonesia–Cipto Mangunkusumo Hospital, Jakarta 10430, Indonesia; 12 Health and Nutrition Science Department, Nutrifood Research Center, PT Nutrifood Indonesia, Jakarta 12930, Indonesia; 13 Department of Biological Sciences, State Islamic University of Sunan Kalijaga (UIN Sunan Kalijaga), Yogyakarta 55281, Indonesia; fahrul.nurkolis.mail@gmail.com

**Keywords:** sulfated polysaccharide from *Caulerpa racemosa*, green algae, obesity-induced cardiometabolic syndrome, gut microbiota modulation, marine algae, antioxidants and biological activity, mTOR-SIRT1-AMPK pathway, PRMT1-DDAH-ADMA

## Abstract

Our investigation intended to analyze the effects of sulfated polysaccharides from *Caulerpa racemosa* (SPCr) in attenuating obesity-induced cardiometabolic syndrome via regulating the protein arginine N-methyltransferase 1-asymmetric dimethylarginine-dimethylarginine dimethylamino-hydrolase (PRMT1-DDAH-ADMA) with the mammalian target of rapamycin-Sirtuin 1–5′ AMP-activated protein kinase (mTOR-SIRT1-AMPK) pathways and gut microbiota modulation. This is a follow-up study that used SPs from previous in vitro studies, consisting of 2,3-di-*O*-methyl-1,4,5-tri-*O*-acetylarabinitol, 2,3,4,6-tetra-*O*-methyl-D-mannopyranose, and type B ulvanobiuronicacid 3-sulfate. A total of forty rats were randomly divided into four treatment groups: Group A received a standard diet; Group B was provided with a diet enriched in cholesterol and fat (CFED); and Groups C and D were given the CFED along with ad libitum water, and daily oral supplementation of 65 or 130 mg/kg of body weight (BW) of SPCr, respectively. Group D showed the lowest low-density lipoprotein, triglyceride, total cholesterol, and blood glucose levels, and the highest HDL level compared to the other groups in this study. These results in the group fed high-dose SPCr demonstrated a significant effect compared to the group fed low-dose SPCr (*p* < 0.0001), as well as in total cholesterol and blood glucose (*p* < 0.05). Supplementation with SPCr was also observed to have an upregulation effect on peroxisome proliferator-activated receptor gamma coactivator (PGC)-1alpha, interleukin 10, Sirtuin 1, DDAH-II, superoxide dismutase (SOD) cardio, and AMPK, which was also followed by a downregulation of PRMT-1, TNF-α, 3-hydroxy-3-methylglutaryl coenzyme A reductase inhibitor, and mTOR. Interestingly, gut microbiota modulation was also observed; feeding the rats with a cholesterol-enriched diet shifted the gut microbiota composition toward the Firmicutes level, lowered the Bacteroidetes level, and increased the Firmicutes level. A dose of 130 mg/kg BW of SPCr is the recommended dose, and investigation still needs to be continued in clinical trials with humans to see its efficacy at an advanced level.

## 1. Introduction

Obesity has emerged as a significant concern for public health in both developed and developing nations. Furthermore, this situation is propagated by the fact that some people are considered “metabolically obese or metabolically unhealthy” even though they have normal weight, which put them at risk for adverse cardiac events [1]. In fact, this phenomenon highlights the correlation between obesity and cardiometabolic risks [2]. Metabolic conditions such as diabetes alter the expression of the protein arginine N-methyltransferase 1-asymmetric dimethylarginine-dimethylarginine dimethylamino-hydrolase (PRMT1-ADMA-DDAH) axis where adversely higher ADMA expression will result in higher PRMT1 and lower DDAH1 expressions [3,4]. It has been suggested that elevated levels of ADMA not only serve as a potential marker for impaired endothelial function but also potentially contribute to the exacerbation of endothelial dysfunction [5]. Interestingly, mammalian target of rapamycin (mTOR), Sirtuin 1 (SIRT1), and 5′ AMP-activated protein kinase (AMPK) have displayed significant roles in energy homeostasis and metabolic stress [6]. A functional food or nutraceutical that is capable of modulating mTOR, SIRT, and AMPK may promote a synergistic beneficial effect in these pathways [7,8].

**Figure 1:**
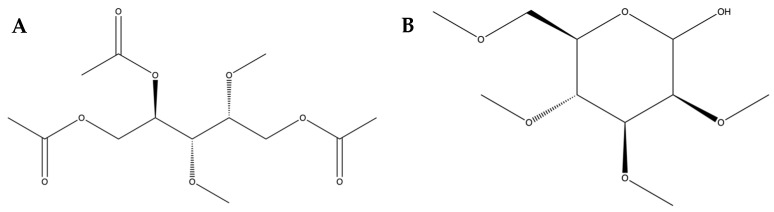
Structural visualization of observed SPs. (**A**) Structural visualization of 2,3-di-*O*-methyl-1,4,5-tri-*O*-acetylarabinitol. (**B**) Structural visualization of 2,3,4,6-tetra-*O*-methyl-D-mannopyranose. Structural visualization of type B ulvanobiuronic acid 3-sulfate cannot be generated using the ChemDraw software version 22.2.0.

In the search for health-benefiting foods, the prowess of marine algae should be considered. They possess a plentiful reserve of various biologically active compounds like polysaccharides, steroids, lipids, polyphenols, and pigments that are known to have properties such as inhibiting tumor growth, fighting against microorganisms, protecting against oxidative damage, preventing ulcers, and promoting wound healing [9]. Sulfated polysaccharides (SPs) from marine algae are also capable of improving obesity-related conditions by attenuating lipid metabolism disorders [10] and suppressing fat accumulation [11]. The authors’ previous work successfully characterized SPs present in *Caulerpa racemosa*, consisting of 2,3-di-*O*-methyl-1,4,5-tri-*O*-acetylarabinitol, 2,3,4,6-tetra-*O*-methyl-D-mannopyranose, and type B ulvanobiuronic acid 3-sulfate (Figure 1) [12].

Even though sulfated polysaccharides are perceived to have considerable health benefits, no research has studied the effect of sulfated polysaccharides from marine algae on cardiometabolic syndrome biomarkers via in vivo study. Therefore, to address the existing information gap, this study aims to assess the effects of sulfated polysaccharides from *Caulerpa racemosa* on obesity-induced cardiometabolic syndrome based on the expression of PRMT1, DDAH, and ADMA in correlation with the mTOR-SIRT1-AMPK pathways and gut microbiota modulation in an in vivo animal model.

Furthermore, this study reports valuable findings to complement current knowledge in the discovery and development of natural-based therapies for metabolic syndrome, particularly for obesity patients. The research presents further data, including the observed correlation of supplementation with natural ingredients, especially SPCr, in modulating the gut microbiome, which has not yet been extensively explored.

## 2. Materials and Methods

### 2.1. Collecting Caulerpa racemosa and Preparing–Isolating Sulfated Polysaccharides from Caulerpa racemosa

The green algae *Caulerpa racemosa* sulfated polysaccharide (SPCr) extracts used in this study were based on the preparation methods described in the authors’ previous study [12]. To be concise, green algae or sea grapes were procured and promptly washed to eliminate any dirt. This process involved cleaning and drying complete green algae at a temperature of 50 °C using an IN55 Memmert incubator oven (Schwabach, Germany), following the protocol established in the previous study. The subsequent step involved grinding the dried specimens to decrease their size. The algae were then macerated for 24 h using an acetone solvent, resulting in the formation of a powdered substance. Furthermore, the characterization of the extracts had been performed in the previously published study [12].

### 2.2. Design of In Vivo Study

#### 2.2.1. Animal Handling Procedures and Ethical Approval

A total of forty male rats (*Rattus norvegicus*), with an average weight of 226.28 ± 12.10 g (3–5 weeks old), were sourced from the Animal Model Farm Yogyakarta, Indonesia, and transported to the research site. The rats were housed in cages under controlled environmental conditions, with a temperature of 27 °C and relative humidity maintained at 50–60%. A balanced light–dark cycle was maintained. A ten-day acclimatization period was provided for all rats before the start of the experiment. Throughout the study, the rats were given unrestricted access to conventional animal feed or pellets provided by PT Citra Ina Feedmill (Jakarta, Indonesia), as well as drinking water. Following the acclimatization period, the rats were randomly divided into four treatment groups. In this animal study conducted in the preclinical stage, the research methodology was designed in accordance with the Declaration of Helsinki and the Council of International Organizations of Medical Sciences (CIOMS). Moreover, all procedures carried out for the animal research complied with the Guidelines for Reporting In Vivo Experiments (ARRIVE), were approved by the Integrated Management Information System of Health Research Ethics—Ethics Committee for Health Research and Development of the Ministry of Health of the Republic of Indonesia—Health Research Ethics Committee of RSUP. Prof. Dr. RD. Kandou with a registration number of 039/EC/KEPK-KANDOU/IV/2023, and received approval from the ethics board of the International Register of Preclinical Trial Animal Studies Protocols (preclinicaltrials.eu) under the registration number PCTE0000370.

#### 2.2.2. Study Design of Treatments

Throughout the duration of the study, experienced veterinarians closely monitored the well-being of the animals, paying attention to any signs of welfare issues, such as reduced food intake, disheveled fur, lethargy, indifference, hiding, or curling up. Weekly examinations were conducted to assess specific health markers and monitor any weight loss in the rats. A total of forty rats were randomly divided into four treatment groups following Federer’s equation, which recommends a minimum sample size of six for an animal experiment involving four groups. Additionally, ten rats were included as backups and for potential additional sample collection purposes. Group A received a standard diet and had access to water ad libitum. Group B was provided with a cholesterol- and fat-enriched diet (CFED) along with unlimited access to water. Groups C and D were given the CFED along with water ad libitum, and daily oral supplementation of 65 or 130 mg/kg of body weight (BW) of *Caulerpa racemosa* sulfated polysaccharides, respectively, was administered by an expert. The daily consumption of animal feed and drinking water was meticulously tracked throughout the entire experiment to ensure there were no differences between the control and experimental groups, and the SPCr extracts were administered by oral gavage.

#### 2.2.3. Composition of Feed and Production of CFED

The composition of the normal pellets used in the study was as follows: 12% moisture, 20% protein, 4% fat, 14% calcium, 1% fiber, 0.7% phosphorus, 11.5% total ash, 0.3% vitamin C, and 0.1% vitamin E. These pellets were sourced from Rat Bio^®^ (Citra Ina Feedmill Ltd., Jakarta, Indonesia). The pellets were stored in a cool and dry environment, away from direct sunlight, in accordance with the manufacturer’s instructions. The CFED was prepared based on previous research [13,14]. The dry, normal pellets were mixed with cholic acid (1%), cholesterol powder (2%), animal fat (20%), and maize oil (2%). After homogenization, distilled water (1 L) was added to the mixer, and the mixture was shaped into smaller pellets. To prevent oxidation, the pellets were dried under sterile conditions at room temperature before being stored at 4 °C. The CFED composition included 43.6% carbohydrates, 12.4% protein, 4.7% fiber, 3.2% fat, 2% cholesterol, 1% cholic acid, 20% animal fat, 4% total ash, 2% maize oil, and the remaining percentage accounted for moisture.

#### 2.2.4. Biomedical Analysis of Blood Samples

Blood samples were collected from the rats six weeks after undergoing interventional feeding. Prior to the blood collection, the animals were fasted overnight and administered ketamine as an anesthetic. Venous sinus blood was drawn into a sterile, dry tube without any anticoagulant, and left to coagulate at room temperature. Venous sinus blood was chosen because it is the site where endothelial dysfunction—which is associated with cardiometabolic syndrome—occurs. After 20 min of centrifugation at 3000 rpm, the serum was separated. Biomedical analyses of low-density lipoprotein (LDL), triglycerides (TG), high-density lipoprotein (HDL), total cholesterol (TC), and blood glucose (BG) were performed using a COBAS Integra^®^ 400 Plus Analyzer (Roche Diagnostics, Basel, Switzerland). Blood samples were also taken from the heart tissue to evaluate additional biomarkers. The activities of the superoxide dismutase (SOD) enzyme were assessed using an SOD Assay Kit from Sigma-Aldrich (Darmstadt, Germany). Serum lipase levels were measured using a Mouse Pancreatic Lipase ELISA Kit (Merck KGaA, Darmstadt, Germany), and serum amylase levels were determined using a Mouse Pancreatic Amylase ELISA Kit (Merck KGaA, Darmstadt, Germany). Inflammatory biomarkers, including peroxisome proliferator-activated receptor-gamma coactivator (PGC)-1alpha (PGC-1α), tumor necrosis factor alpha (TNF-α), and interleukin (IL-10), were quantified using specific ELISA kits. A PGC-1α Mouse ELISA Kit (Sunlong Biotech Co., Ltd.; Hangzhou, China) was used for PGC-1α, a Mouse Tumor Necrosis Factor-α (TNF-α) Kit (Sunlong Biotech Co., Ltd.; Hangzhou, China) was used for TNF-α, and an IL-10 ELISA Kit (Abcam, Cambridge, UK) was used for IL-10. The cardiometabolic biomarkers PRMT-1 and DDAH-II were quantified using ELISA kits from LSBio (Seattle, WA, USA). PRMT-1 was assessed using an LS-F65223 ELISA Kit, and DDAH-II was measured with an LS-F14238 ELISA Kit. ADMA levels were directly evaluated using an ALX-850-327-KI01 ADMA Direct Mouse ELISA Kit from Enzo Life Sciences, Inc. (New York, NY, USA). The body weights of the rats were determined using digital scales.

### 2.3. The Sequencing and Analysis of the 16S rRNA Gene in Rat Feces for Gut Microbiota

Stool samples were stored at −80 °C before microbiome gut analysis was performed. Intestinal bacterial genomes were extracted from the fecal samples using OMG soil extraction kits from Shanghai Meiji Biopharmaceutical Technology Co., Ltd. (Shanghai, China). The statistical analysis allowed the researchers to select feces from the normal, CFED only, and high dose of SPCr groups for gut microbiome analysis. The V3-V4 variable region of the 16S rRNA gene was amplified through polymerase chain reaction (PCR) using the primers 338F (5′-ACTCCTACGGGAGGCAGCAG-3′) and 806R (5′-GGACTACHVGGGTWTCTAAT-3′). Sequencing was performed on the Illumina Miseq PE300 platform. The raw sequences were processed using the FAST software version 3.5.2, trimmed using the FLASH software, and high-quality reads were grouped into Operational Taxonomic Units (OTUs) with 97% similarity using the UPARSE software version 7.1. Chimera sequences were eliminated using the search software. Each sequence was taxonomically classified at the species level using the ribosomal database project (RDP) classification, with a comparison threshold of 70% against the Silva 16S rRNA database (Version 138 by Max Planck Institute for Marine Microbiology and Jacobs University, Bremen, Germany).

### 2.4. Data Analysis and Management

Multivariate ANOVA was employed to analyze various parameters, including LDL, HDL, TG, TC, and BG (lipid profile) for lipid profile; IL-10, TNF-α, and PGC-1α as inflammatory biomarkers; SOD cardio, serum lipase, and serum amylase (enzymatic assays); and PRMT-1, DDAH-II, and ADMA (cardiometabolic biomarkers) in an in vivo setting. Paired *t*-tests or dependent *t*-tests were utilized to determine significant differences between initial and final body weights for each group. One-way ANOVA was conducted to assess disparities in secondary parameters, such as water intake (mL), food intake (g), food efficiency ratio (FER), and initial, final, and daily weight gain (g/day) across the groups. Mean values ± standard error of the mean (SEM) were presented with a 95% confidence level. The GraphPad Prism version 9.4.1 software (Boston, MA, USA) was employed for both in vitro and in vivo data analyses using a MacBook. Gut microbiota analysis was performed using MicrobiomeAnalyst version 2.0.

## 3. Results

### 3.1. Effects of Sulfated Polysaccharides of Caulerpa racemosa on the Cardiometabolic Biomarkers in Rats

The data on the body weight, feed, water intake, and food efficiency ratio (FER) characteristics of the experimental rats are presented in Table 1. The initial parameters of body weight did not differ significantly across all groups (*p* > 0.05), and neither did the parameters differ across intervention (weight gain, food intake, water intake, and FER). Furthermore, the body weight of the rats changed significantly through the intervention (*p* < 0.05), with the highest BW observed in Group B (CFED only) and the lowest one observed in Group D (CFED + High Dose (HD) of SPCr). It was noteworthy that the final BW of the rats showed a significant difference between groups (*p* < 0.0001), implying that different interventions yielded distinctive effects.

**Figure 2:**
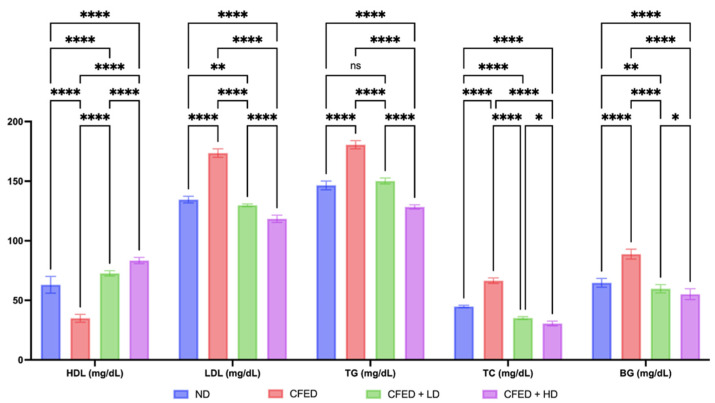
Effect of sulfated polysaccharides of *Caulerpa racemosa* on blood glucose and lipid profile in rats. High-density lipoprotein–HDL; low-density lipoprotein–LDL; triglycerides–TG; total cholesterol–TC; blood glucose–BG. **** *p* < 0.0001, ** *p* = 0.0070, * *p* = 0.0123, and ns *p* > 0.05.

Furthermore, the group that was only given the CFED had a higher final BW value compared to the normal group and the intervention group with SPCr. Interestingly, the CFED group given the SPCr intervention had even lower final BW values compared to the normal group. In addition, improvements in blood glucose and lipid profile markers were observed, as shown in Figure 2.

**Figure 3:**
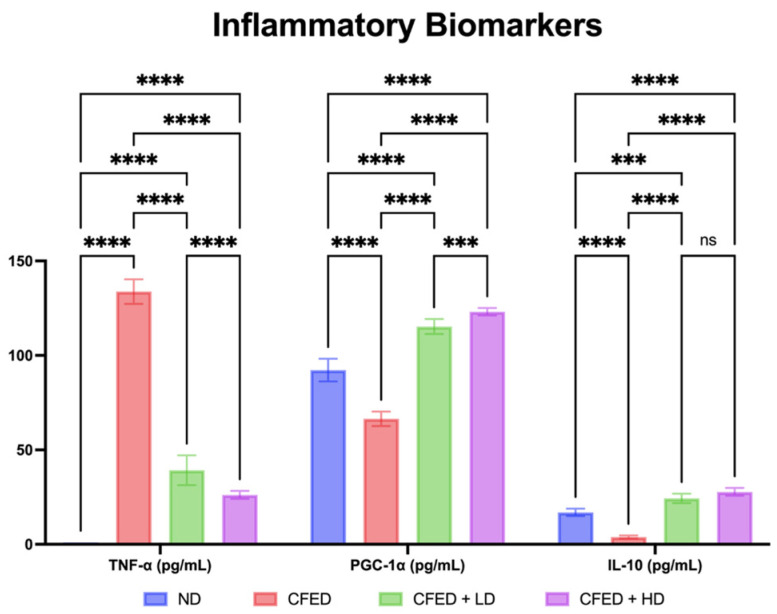
Effect of sulfated polysaccharides of *Caulerpa racemosa* on inflammatory markers in rats: peroxisome proliferator-activated receptor gamma coactivator (PGC)-1alpha–PGC-1α; tumor necrosis factor-alpha–TNF-α; and interleukin 10–IL-10. **** *p* < 0.0001, *** *p* = 0.0004, and ns *p* > 0.05 (*p* = 0.2225).

The CFED clearly appears to have a significant metabolic effect, as indicated by an increase in LDL, TG, TC, and BG, which was followed by a decrease in HDL. The CFED + HD group resulted in the lowest LDL, TG, TC, and BG, and the highest HDL compared to other groups in this study (Figure 2). The result in the high-dose SPCr group demonstrated a significant effect compared to low-dose SPCr (*p* < 0.0001), as well as in total cholesterol and blood glucose (*p* < 0.05). In addition, improvements in inflammatory markers were also observed, and the data are presented in Figure 3.

**Figure 4:**
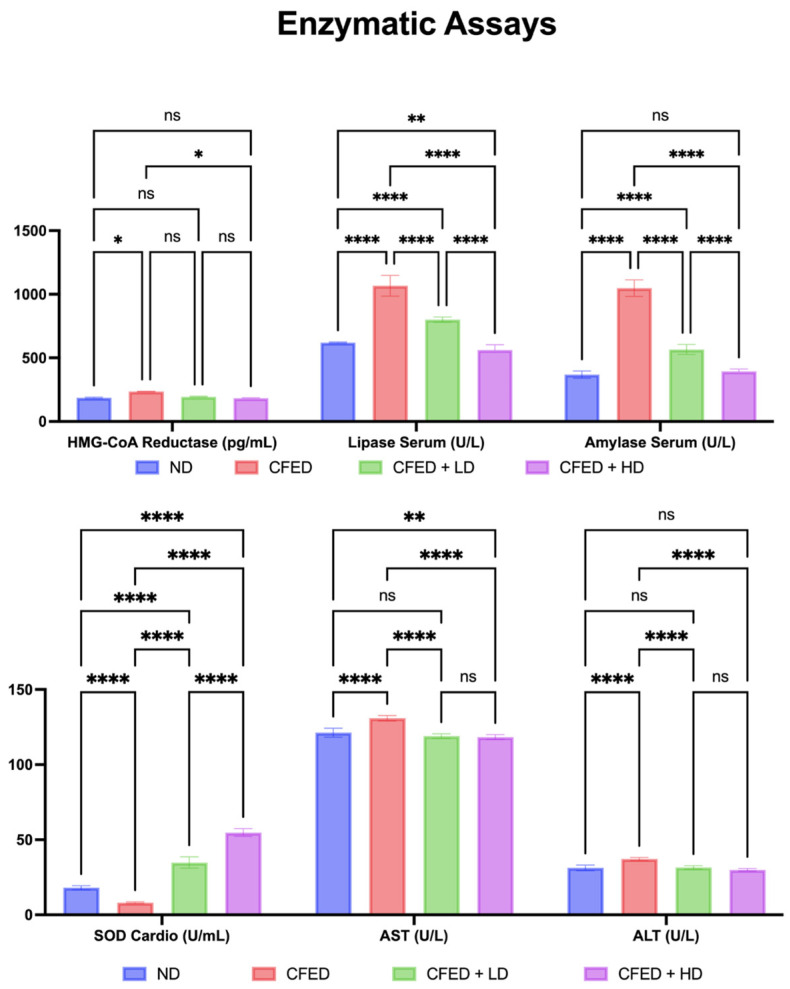
Effect of sulfated polysaccharides of *Caulerpa racemosa* on serum enzyme levels in rats: 3-hydroxy-3-methylglutaryl coenzyme A (HMG-CoA) reductase inhibitor; superoxide dismutase (SOD) cardio; aspartate transaminase (AST); and alanine transaminase (ALT). **** *p* < 0.0001, ** *p* = 0.0026, * *p* = 0.0165, and ns *p* > 0.05.

The group of CFED + HD resulted in the highest PGC-1α and IL-10 and the lowest TNF-α among all groups (Figure 3). This result showed significant difference in the analysis of TNF-α and PGC-1α, but not in the analysis of IL-10. The improvement in inflammatory markers is consistent with the effect of SPCr on the serum enzyme levels in the rats, which is presented in Figure 4.

**Figure 5:**
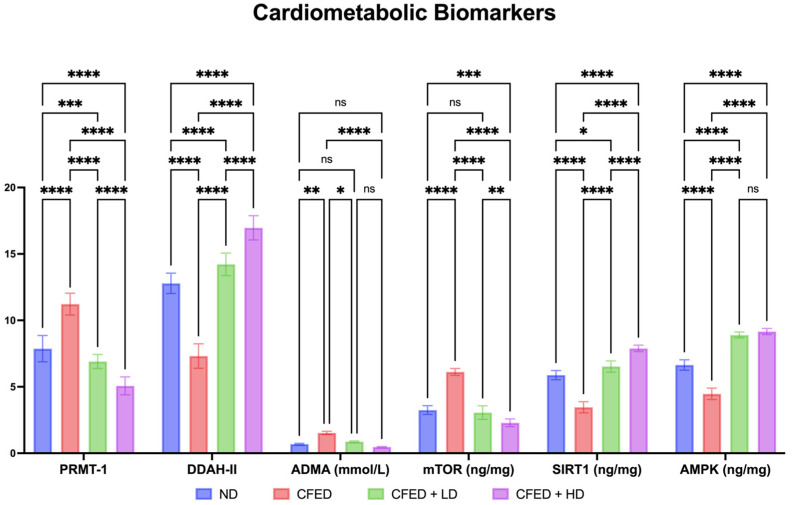
Effect of sulfated polysaccharides of *Caulerpa racemosa* on PRMT-1/DDAH/ADMA and mTOR/SIRT1/AMPK expressions in rats: protein arginine N-methyltransferase 1 (PRMT-1); dimethylarginine dimethylamino-hydrolase 2 (DDAH-II); plasma asymmetric dimethylarginine (ADMA); mammalian target of rapamycin (mTOR); Sirtuin 1 (SIRT1); and 5′ AMP-activated protein kinase (AMPK). **** *p* < 0.0001, *** *p* = 0.0005, ** *p* = 0.0094, * *p* = 0.0404, and ns *p* > 0.05.

The CFED + HD group significantly demonstrated a reduction in lipase serum and amylase serum and an increase in SOD cardio serum. However, the supplementation of SPCr did not have a significant effect on 3-hydroxy-3-methylglutaryl coenzyme A (HMG-CoA) reductase inhibitor, AST, and ALT levels (Figure 4). However, it is clear that the CFED has an increased effect on HMG-CoA reductase inhibitors compared to the normal controls. More interestingly, the CFED + HD group significantly demonstrated the effect of this intervention in reducing HMG-CoA reductase, implying the potential as an HMG-CoA reductase inhibition. The effect of SPCr in improving serum enzyme levels in the rats is also supported by the results on cardiometabolic biomarkers shown in Figure 5.

These results show that PRMT-1 and mTOR are significantly reduced in the group of CFED + HD. In addition, SIRT1 and DDAH-II are significantly increased in the group of CFED + HD. However, in terms of ADMA, SPCr does not have a significant effect. In addition, SPCr supplementation results in a significant increase in AMPK level, which is associated with the dose used, and there is no significant correlation between LD and HD (Figure 5).

### 3.2. Modulation of Gut Microbiome in Rats Fed with Cholesterol- and Fat-Enriched Diet Supplemented with Sulfated Polysaccharides of Caulerpa racemosa

#### 3.2.1. Effect of Sulfated Polysaccharides of *Caulerpa racemosa* on Gut Microbiota Composition

**Figure 6:**
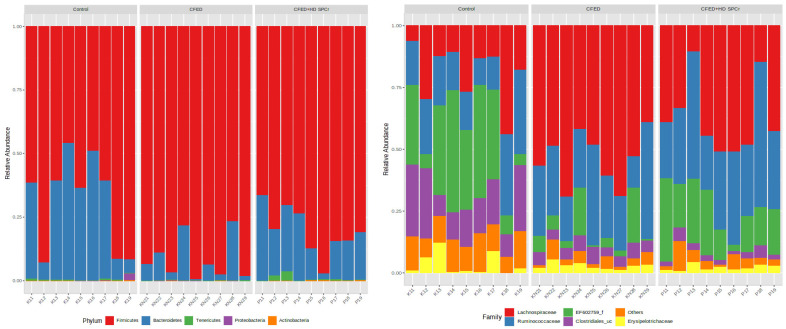
Representation of the taxonomic composition is displayed at the genus level (on the **left**) or phylum level (on the **right**). The colored bars indicate the proportional abundance of each phylum or genus in relation to the total microorganisms.

The composition of gut microbiota in the rats is presented in Figure 6. At the phylum level, the gut microbiota of the control rats was composed mainly of Firmicutes and Bacteroidetes. Feeding the rats with a cholesterol-enriched diet shifted the gut microbiota composition toward Firmicutes and lowered the Bacteroidetes level. Supplementation with sulfated polysaccharides of green algae increased the Bacteroidetes at the phylum level. At the family level, it was confirmed that supplementation with sulfated polysaccharides increased the EF602759 compared to the cholesterol-enriched diet group.

#### 3.2.2. Effect of Sulfated Polysaccharides of *Caulerpa racemosa* on Gut Microbiota Diversity

**Figure 7:**
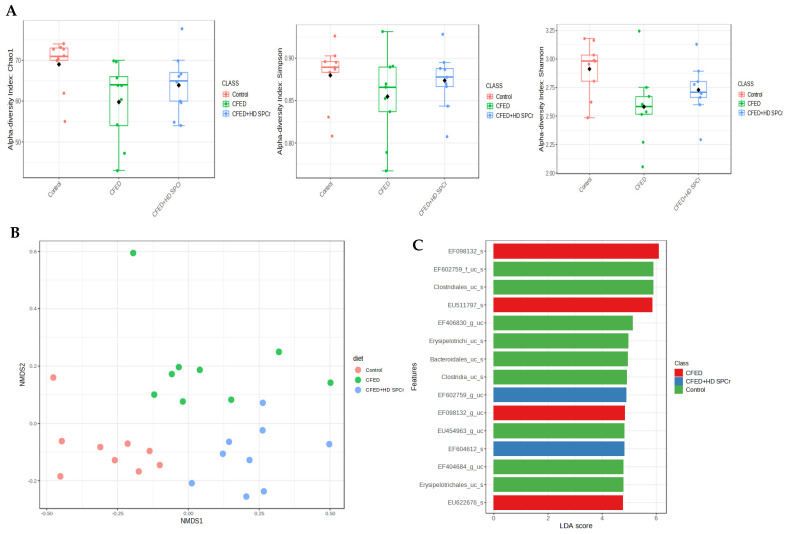
Effect of sulfated polysaccharides of green algae (*Caulerpa racemosa*) on gut microbiota diversity. (**A**) Alpha diversity level of gut microbiota based on Chao1, Shannon, and Simpson indexes. The cholesterol- and fat-enriched diet (CFED) has significantly lower diversity compared to other groups. (**B**) Non-metric multidimensional scaling (NMDS) plot of beta diversity using Bray–Curtis distance matrix. Each point represents the gut microbiota among the groups (control, CFED, or CFED + HD SPCr). The gut microbiota with a similar composition tend to cluster together in the same area of the graph, while dissimilar gut microbiota composition points away from each other. (**C**) Linear discriminant analysis (LDA) score of the significant discriminant taxa among the groups (LDA score > 4.0, FDR *p* < 0.05). Control (normal) rats (n = 10), cholesterol-enriched diet/CFED (n = 10), and cholesterol-enriched diet supplemented with 45 mg/kg BW of sulfated polysaccharides of green algae/CFED + HD SPCr (n = 10).

The alpha diversity analysis (Chao1, Shannon, and Simpson indexes) showed a significant (*p* < 0.05, Kruskal–Wallis test) difference among the three groups, and the results are presented in Figure 7. The rats fed a cholesterol-enriched diet particularly had the lowest average diversity value compared to the other groups. Feeding rats with sulfated polysaccharides increased the diversity of gut microbiota when the rats were put on a cholesterol-enriched diet.

The non-matrix multidimensional analysis (NMDS) was performed to analyze the beta diversity of the gut microbiota. The result showed that the stress for the NMDS analysis was 0.12. The PERMANOVA analysis based on the Bray–Curtis matrix showed that the composition of gut microbiota was statistically different among the three groups (*p*-value = 0.001, R2 = 0.283). A hierarchical clustering using the Ward algorithm on the Bray–Curtis distance matrix was performed to identify which groups were clustered together. The results showed that the group fed the cholesterol- and fat-enriched diet (CFED) was clustered together with the groups fed green algae sulfated polysaccharides, while the control group was in a separate cluster.

**Figure 8:**
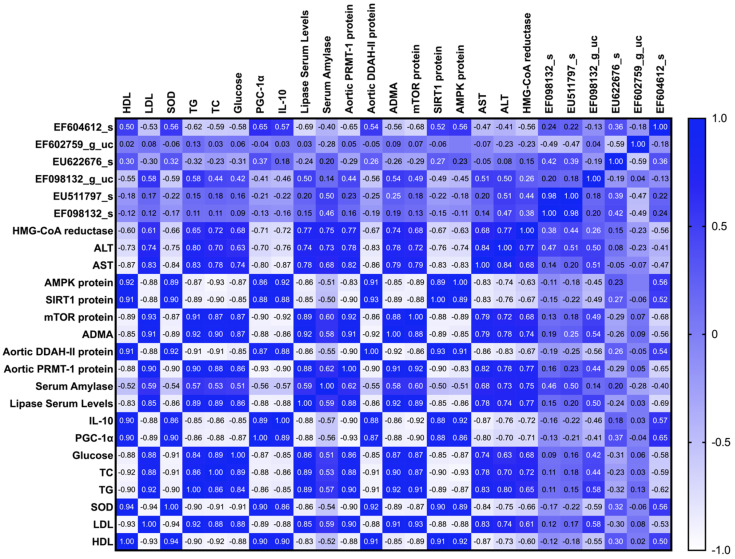
Heatmap of Pearson correlation between the gut microbiome and cardiometabolic biomarkers in rats.

Next, to analyze for significantly different taxa among the groups, linear discriminant analysis (LEfSe) was performed. Using an LDA score of 4.0 with an FDR-adjusted value of 0.05, there were 22 significantly different taxa among the groups. The group fed with the cholesterol-enriched diet was enriched with EF098132, EU511797, and EU622676 bacteria, which are Firmicutes bacteria. Bacteria EF602759 and EF604612 were enriched in the groups fed green algae sulfated polysaccharides. The results of the LEfSe analysis are presented in Figure 7. To further analyze whether there is a correlation between gut microbiome and cardiometabolic biomarkers in the rats, we performed Pearson correlation test, and the data are presented in Figure 8.

The population of EF604612_s, which was enriched in the group fed sulfated polysaccharides of green algae, has a significant (*p* < 0.05) negative correlation with serum LDL, triglyceride, cholesterol, glucose, lipase, amylase, aortic PRMT-1 protein, ADMA, mTOR protein, AST, ALT, and HMG-CoA reductase. This bacterium also has a significant (*p* < 0.05) positive correlation with serum HDL, SOD, PGC1α, IL-10, aortic DDAH-II protein, SIRT1, and AMPK protein. However, a reverse trend in correlation was observed for EF098132_g_uc, the family of Lachnospiraceae of Firmicutes, which was enriched in the group fed the CFED.

## 4. Discussion

The risk of developing metabolic abnormalities, insulin resistance, and cardiovascular illnesses has increased considerably as obesity has become a global health concern [15]. For the creation of efficient therapeutic approaches, addressing the fundamental processes underlying the cardiometabolic syndrome associated with obesity is essential. To address the cardiometabolic syndrome caused by obesity, this study goes deeply into understanding the complex interactions between numerous biochemical pathways and gut bacteria. The function of sulfated polysaccharide derived from the marine green alga *Caulerpa racemosa*, which is well-known for its potential medicinal benefits, was studied. This research demonstrated how this polysaccharide can control the gut microbiota, mTOR-SIRT1-AMPK networks, and PRMT1-DDAH-ADMA pathways, all of which are involved in the pathophysiology of cardiometabolic syndrome. The main conclusions of this study—with its significance for future investigations and treatment approaches—could be the setting stone for understanding the potential therapeutic uses of such intervention as well as the difficulties that lie ahead in developing efficient medicines for people with cardiometabolic syndrome associated with obesity.

In this study, in terms of blood glucose and lipid profiles, the groups that received the CFED and low or high doses of *Caulerpa racemosa* SPs showed meaningful decreases in LDL, TG, TC, and BG, coupled with an increase in HDL (*p* < 0.05), compared to the group that received the CFED only. The decrease in lipid profile markers and blood glucose was also accompanied by a decrease in weight in the SPCr-intervened rats. The reduction in LDL, TG, TC, and BG is thought to contribute strongly to the reduction in fat storage in many organs and or tissues, which can be seen from the resulting weight loss [16]. Due to their structural versatility, natural polysaccharides, including biopolymers such as proteins and nucleic acids, possess remarkable biological properties. Polysaccharides derived from natural sources, particularly sulfated polysaccharides, have demonstrated significant effects in reducing lipid and cholesterol levels. These effects are achieved through various mechanisms, such as antioxidative and anti-inflammatory activities, elevation in high-density lipoprotein (HDL) levels, reduction in free fatty acids, suppression of enzymes like HMG-CoA reductase and CYP7A1, modulation of SREBP1, promotion of bile acid secretion, and lowering of TC, TG, VLDL, and LDL levels, which may contribute to weight loss and obesity risk [16]. As for blood glucose level, both laboratory settings and live animal experiments have demonstrated that administering these polysaccharides leads to hypoglycemic, hypolipidemic, antioxidant, and anti-inflammatory effects through a contributed increase in pancreatic β-cell mass and improvement in β-cell function. These polysaccharides also enhance insulin signaling pathways by acting on insulin receptors and activating the PI3K/Akt pathway, ultimately regulating the ERK/JNK/MAPK pathway [17]. Furthermore, type B ulvanobiuronicacid 3-sulfate—a member of polysaccharide ulvan—was also characterized and might contribute to the beneficial health properties of *Caulerpa racemosa* SPs [12]. In conjunction with that, the authors’ previous work demonstrated that *Caulerpa racemosa* SPs have anti-obesity, antiaging, antidiabetic, and antioxidant properties [12,18].

The anti-inflammatory activities of *Caulerpa racemosa* SPs were also studied. PGC-1α levels were found to be higher in the CFED + HD group, which may indicate better oxidative metabolism and activation of mitochondrial biogenesis. It is well recognized that PGC-1α is essential for controlling mitochondrial activity and energy metabolism [19]. The increased levels of PGC-1α in this group suggest a possible improvement in the cellular mechanisms for generating and using energy. Additionally, the CFED + HD group’s increased levels of the anti-inflammatory cytokine IL-10 point to a potential decrease in inflammation. The immunosuppressive effects of IL-10 and its capacity to reduce inflammatory responses are well recognized [20]. A modification of the immune system toward an anti-inflammatory state may be indicated by the elevated IL-10 levels in the CFED + HD group. This might have positive impacts on general health and disease prevention. However, it is crucial to remember that there was no statistically significant difference in IL-10 levels across the groups. The sample size, inter-individual variability, or the dose of the intervention are a few possible causes of this lack of significance. TNF-α, a cytokine that promotes inflammation and is linked to several inflammatory disorders [21], was present in the lowest amount in the CFED + HD group. The decrease in TNF-α levels raises the possibility that the CFED + HD therapy may have an anti-inflammatory impact, which is in line with another study [22]. These data support the concept that the therapy may have an anti-inflammatory impact as they correlate with the observed rise in IL-10 levels, as also observed by [23,24].

The CFED + HD group’s lower levels of amylase and lipase blood levels represent the anti-obesity and antidiabetic capabilities of *Caulerpa racemosa* SPs. Inhibition of amylase prolongs the duration of carbohydrate digestion, which subsequently lowers postprandial glucose and hyperglycemia [25]. The supplementation of SPs acts as a lipase inhibitor, which plays a role in offsetting obesity by preventing excessive fat deposition through lipolysis [26]. Additionally, the CFED + HD group’s higher levels of the antioxidant enzyme SOD point to improved antioxidant protection. Scavenging ROS and shielding cells from oxidative stress is an important function of SOD, which sets up antioxidant defense systems that prevent cardiometabolic diseases [27]. The increased SOD levels suggest that the CFED + HD therapy may help prevent oxidative damage and improve cellular health. These results correspond to the findings of previous in vitro work that *Caulerpa racemosa* SPs have anti-obesity, antidiabetic, and antioxidant properties [12], but the results may be different in further in vivo tests. The liver primarily regulates the synthesis of cholesterol through the activity of a key enzyme known as HMG-CoA reductase. This enzyme plays a crucial role in converting HMG-CoA into mevalonic acid. Inhibiting the activity of HMG-CoA reductase, an enzyme involved in cholesterol production, can assist in controlling cholesterol levels [28]. SPCr may not directly affect HMG-CoA reductase activity, as evidenced by the lack of significant differences between the treatments. Furthermore, supplementing with SPCr had no discernible effect on the levels of AST and ALT, two indicators of liver function. Lower AST and ALT levels have been associated with increased cardiometabolic risks [29]. This finding is similar to another in vivo study in which SP treatment resulted in reduced histopathological damage, decreased expression of CYP2E1, lowered levels of serum AST and ALT, and improved antioxidant and anti-inflammatory properties [30].

The CFED + HD group’s much lower levels of PRMT-1, mTOR, and ADMA indicate that this intervention has an inhibitory effect on these proteins. The dysregulation of PRMT-1, which is in charge of methylating arginine residues, has been linked to several illnesses such as disorders of cardiovascular function [31]. The drop in PRMT-1 levels suggests that arginine methylation patterns may have been altered by the CFED + HD therapy. The downregulation of mTOR, a crucial regulator of cell growth and metabolism, in the CFED + HD group raises the possibility that cellular signaling pathways involved in growth and metabolism have been altered [32]. On the one hand, the significantly higher levels of SIRT1 and DDAH-II in the CFED + HD group imply that the therapy upregulates these proteins. An NAD+-dependent deacetylase called SIRT1 is involved in several physiological functions, such as metabolism, stress response, and aging [33]. The elevated levels of SIRT1 suggest that the cellular pathways involved in these activities may have been activated. On the other hand, ADMA—a chemical connected to cardiovascular health—is processed by the enzyme DDAH-II. The increased levels of DDAH-II imply a better ability to metabolize ADMA, which may improve cardiovascular function [34]. It is interesting to see that SPs had no discernible impact on ADMA levels. Nitric oxide synthase is known to be inhibited by ADMA, which also causes endothelial dysfunction. Although the lack of an impact on ADMA levels implies that SPCr may not have a direct impact on its metabolism, it is vital to take into account other possible processes by which SPCr may have an impact on cardiovascular health. Additionally, the SP supplementation led to a notable rise in AMPK levels. Cellular energy homeostasis is significantly regulated by AMPK, which is also engaged in many metabolic processes [35]. The increased AMPK levels indicate that energy-regulating mechanisms are activated in response to the SPCr administration, which may result in better metabolic outcomes. Regarding dosage, there was no discernible difference in the AMPK levels between the LD and HD supplementation. This may imply that there was no dose-dependent response in AMPK activation for the dosage range examined in this investigation. The ideal dose for AMPK regulation may require further investigation of various dosing methods.

A novel molecular study of SPCr originating from natural substances to combat metabolic syndrome is highly warranted, particularly in treating dyslipidemia, diabetes mellitus, obesity, and cardiovascular diseases. The focus on developing in vivo studies is crucial to demonstrate its potential impact in future clinical practice [36]. The findings of this SPCr study align with other research showing anti-metabolic syndrome activities, indicating that SPCr could serve as an alternative antidiabetic agent not only in lowering glycemia but also in reducing cardiovascular risk through complex mechanisms, being potentially more potent than metformin monotherapy [37]. Moreover, the strong antioxidant activity of SPCr is believed to contribute to attenuating obesity-induced cardiometabolic syndrome in animal models, as described in a review study highlighting the potential of antioxidant therapy with significant cardiovascular risk benefits [38]. Interestingly, this study also observed AMPK activation, linking it to other studies proposing AMPK as a potential activator or a new therapy for diabetes and related complications like cardiometabolic syndrome [39]. Given the potential of SPCr in attenuating metabolic syndrome, further specialized development is necessary, particularly in conducting advanced human clinical trials. Furthermore, the combination of nanomaterials sciences with SPCr holds promise for developing new, advanced techniques for targeting metabolic syndrome [40]. Such a technique holds the potential to serve as a more precise carrier of SP molecules targeting specific targets.

EF604612_s and EU622676_s strains are beneficial for HDL while also improving other blood lipid profiles and blood glucose. The same strains also increase the expression of PGC-1α and IL-10. Only EF604612_s is strongly linked with a reduction in serum amylase and lipase levels. EF604612_s and EU622676_s strains have a negative relationship with PRMT-1, ADMA, and mTOR parameters. The same strains also elevate the expression of DDAH-II, SIRT1, and AMPK proteins. Interestingly, EF604612_s and EF602759_g_uc strains lower the expression of HMG-CoA, AST, and ALT.

As a strength, this study represents the first successful investigation of sulfated polysaccharides in regulating the PRMT1-DDAH-ADMA with mTOR-SIRT1-AMPK pathways and gut microbiota modulation. The antioxidant activity and in vitro biological activities observed in previous studies are further supported by the in vivo results of this study. Moreover, a noteworthy finding of this research is the observed correlation between gut microbiome and cardiometabolic biomarkers in rats, providing new insights for the comprehensive development of metabolic syndrome therapeutics. However, it is important to note that this study is currently limited to animal models and cannot directly translate to human trials. Hence, further research involving human clinical trials is highly warranted, and the dosages used in this study can serve as a reference for such clinical investigations in the future.

## 5. Conclusions

Dietary supplementation of sulfated polysaccharides from *Caulerpa racemosa* (SPCr) might be an alternate treatment to improve lipid profiles, inflammatory biomarkers, and metabolic syndrome-related enzyme serum levels dramatically. Interestingly, gut microbiota modulation was also observed. A new concept was explored: feeding rats with a cholesterol-enriched diet shifted the gut microbiota composition toward Firmicutes level, lowered the Bacteroidetes level, and increased the Firmicutes level. Finally, dietary supplementation with a high dose of SPCr (2,3-di-*O*-methyl-1,4,5-tri-*O*-acetylarabinitol, 2,3,4,6-tetra-*O*-methyl-D-mannopyranose, and type B ulvanobiuronicacid 3-sulfate) attenuates the obesity-induced cardiometabolic syndrome via regulating the PRMT1-DDAH-ADMA with mTOR-SIRT1-AMPK pathways and gut microbiota modulation.

## 6. Patents

The SP preparation method with its formulation resulting from the work reported in this article has been registered as a patent with the number S00202301037 (Fahrul Nurkolis is the patent holder) in Indonesia.

## Figures and Tables

**Table 1:** Body weight, feed, water intake, and FER characteristics of experimental rats (*Rattus norvegicus*).

Group	Normal	CFED	CFED + Low Dose SPCr	CFED + High Dose SPCr	*p* **
Initial BW (g)	224.62 ± 10.75	227.11 ± 15.46	227.19 ± 12.94	226.20 ± 10.39	0.7691
Final BW (g)	256.93 ± 4.97	277.37 ± 7.22	244.78 ± 4.03	242.68 ± 3.49	<0.0001
*p* *	<0.000001	0.000002	0.000438	0.001952	
Weight gain (g/day)	0.7 ± 0.20	1.09 ± 0.29	0.38 ± 0.28	0.36 ± 0.21	0.9989
Food intake (g)	5.43 ± 0.32	4.93 ± 0.72	5.13 ± 1.12	5.10 ± 0.48	0.9976
Water intake (mL)	5.81 ± 0.56	5.74 ± 0.87	5.35 ± 1.00	5.21 ± 0.53	0.9971
FER (%)	13.13 ± 4.25	22.37 ± 6.46	7.39 ± 5.61	7.17 ± 4.35	0.0035

* Dependent or paired *t*-test 95% CI (0.05). ** ANOVA 95% CI (0.05). Food efficiency ratio (FER) was calculated by dividing body weight (BW) gain by food intake. Cholesterol- and fat-enriched diet (CFED); sulfated polysaccharides from *Caulerpa racemosa* (SPCr).

## Data Availability

The datasets presented in this study can be requested from the corresponding author.
